# An Improved Step-Type Liquid Level Sensing System for Bridge Structural Dynamic Deflection Monitoring

**DOI:** 10.3390/s19092155

**Published:** 2019-05-09

**Authors:** Xijun Ye, Zhuo Sun, Xu Cai, Liu Mei

**Affiliations:** 1 School of Civil Engineering, Guangzhou University, Guangzhou 510006, China; xijun_ye@gzhu.edu.cn (X.Y.); zhuosun.gzhu@gmail.com (Z.S.); 2 Guangdong Provincial Key Laboratory of Durability for Marine Civil Engineering, Shenzhen University, Shenzhen 518060, China; meiliu@szu.edu.cn

**Keywords:** structural health monitoring, dynamic deflection, liquid level sensing system, step-type

## Abstract

Real-time and accurate monitoring of dynamic deflection is of great significance for health monitoring and condition assessment of bridge structures. This paper proposes an improved step-type liquid level sensing system (LLSS) for dynamic deflection monitoring. Layout of straight-line-type pipeline is replaced by step-type pipeline in this improved deflection monitoring system, which can remove the interference of the inclination angle on the measurement accuracy and is applicable for dynamic deflection monitoring. Fluid dynamics are first analyzed to demonstrate that measurement accuracy is interfered with by the fluid velocity induced by structural vibration, and ANSYS-FLOTRAN is applied for analyzing the influence range caused by the turbulent flow. Finally, a step-type LLSS model is designed and experimented with to verify the influence of the three key parameters (initial displacement excitation, step height, and distance from the measurement point to the elbow) on the measurement accuracy, and the reasonable placement scheme for the measurement point is determined. The results show that the measurement accuracy mainly depends on the turbulent flow caused by step height. The measurement error gets smaller after about 1.0 m distance from the elbow. To ensure that the measurement error is less than 6%, the distance between the measurement point and the elbow should be larger than 1.0 m.

## 1. Introduction

### 1.1. Background

Bridge structures are important components of the transportation network of highways and urban areas. They are designed and built to be safe against failure and to perform satisfactorily over their service life [1]. However, the traffic volume has increased significantly with the rapid development of the economy and transportation, and overloading has become a more serious threat in recent years. Bridge structures are continuously deteriorating due to misuse, material ageing, and inadequate maintenance [2]. Therefore, structural health monitoring (SHM) of bridge structures is necessary to ensure both their safety and serviceability over their lifespan. Excessive deformations, particularly dynamic deflection under varying environmental conditions and repeated moving loads due to traffic, is one of the key indices in the application of SHM, which can directly reflect the safety and serviceability of a bridge structure. Gradual or sudden changes in the service load deflections are indicative of structural changes, damage, or deterioration [3].

Over the past few decades, a number of methods have been developed for deflection measurement, and they are classified into contact-type and contactless-type [4]. Contact-type methods include linear variable differential transformers (LVDTs) [5], global positioning systems (GPS) [6], inclinometers [7], gyroscopes [8], accelerometers, and liquid level sensing systems (LLSS) [9]. The principle of the contact-type method is that the measurement points need to be placed on the structure, and a fixed measurement point has to be chosen as the reference point to measure the relative deflection between other measurement points and the reference point. However, each of the contact-type methods has its limitation in field applications. For example, for real-time monitoring of deflections under operating traffic loads using LVDT, it is difficult to establish a fixed reference point for displacement transducers when bridges are over rivers, seas, or have high clearances [5,10]. The measurement accuracy for dynamic deflection monitoring of GPS is at the centimeter level. Due to their high maintenance costs and low measurement accuracy levels, they are often applied to cable-stayed bridges or suspension bridges [6,11,12,13]. Inclinometers are easy to install and maintain but have high requirements for hardware [7,14,15]. The application of a traditional gyroscope is limited by high cost, large size, and low precision. Gyroscope drift will also cause a large attitude error in a short time [8]. Theoretically, displacements can be obtained by implementation of numerical integration twice on the measured acceleration data, but drift error and direct-current (DC) bias will be amplified in the numerical integration procedure [16]. LLSS has been widely used for static deflection measurements and has demonstrated a high accuracy (errors less than 0.1 mm). However, in field applications, both the varying environmental conditions and system errors during installation (e.g., air bubbles in the pipeline, leakages) will disturb the precision [9,17].

To overcome the limitations of the contact-type, deflection measurement technology is developing towards the contactless-type, which can measure the three-dimensional (3D) full-field dynamic deflection. Contactless-type methods include vision-based monitoring systems, motion capture systems (MCS), and active 3D sensors. With the development of photographic video tracing and image processing technology, vision-based monitoring systems, such as laser scanners, laser trackers, and digital cameras, have been developed as an effective alternative method for deflection monitoring [18]. The previous studies showed that some complex algorithms are needed to derive the actual displacement in vision-based techniques, and the precision can be interfered with by foggy or rainy weather [18,19,20,21,22]. The 3D movements of markers, which are deployed on bridges or buildings, can be captured by MCS with high accuracy and high sampling frequency. In civil engineering applications, MCS had been used for position measurement of structures under dynamic excitation [23,24,25]. Three-dimensional sensing devices (e.g., Microsoft Kinect) were originally developed for entertainment purposes but can recognize hand gestures and the human body. This state-of-the-art technology has attracted attention for applications in dynamic deflection-field measurement [4,26,27]. For outdoor application, the measurement accuracy is restricted by system limitations (including dynamic scenery, ambient background light, multi-sensor interference, scattering media, and semi-transparent, etc.), and 3D sensing technology is still advancing to overcome the system limitations [28,29,30,31,32].

### 1.2. Motivation and Objectives

For a deflection monitoring system, the main technical requirements are consistency, accuracy, ease of installation, stability, and high cost-effective performance. Despite numerous emerging deflection measurement techniques, one of the most successful methods for short-term and long-term deflection measurement of bridge structures is the LLSS. Although there are some factors interfering with the measurement accuracy in the process of installation and measurement, with adequate conception and installation precautions, the causes of the errors can be controlled and minimized [33]. This method has been used for more than 40 years, and the applications of static deflection measurement for large-scale bridges have proved its validity [34,35]. However, few studies on dynamic deflection measurement use LLSS. Some researchers have conducted preliminary studies on the oscillation characteristics of liquid levels and the correlation between the liquid pressure and structural deflection [36,37].

**Figure 1:**
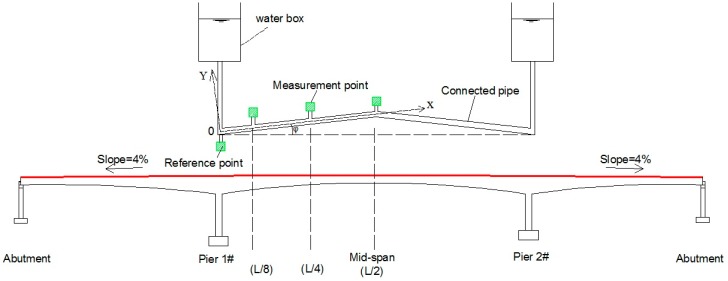
Layout of the straight-line-type liquid level sensing system (LLSS).

In Figure 1, the longitudinal slope (0–5%) is usually designed in long-span bridges, which would create a height difference between the abutment and mid-span. Therefore, an inclination angle (*φ*) is introduced while the pipeline is deployed in a straight-line type. Our previous work [36,37] focused on the application of dynamic deflection measurement using LLSS and showed that the deflection measurement accuracy is mainly interfered with by two factors: the additional pressure of the fluid caused by structural vibration, and the inclination angle of the pipeline. This study develops a step-type LLSS with high measurement accuracy for dynamic deflection monitoring. The layout of a straight-line-type pipeline is replaced by a step-type pipeline in this improved deflection monitoring system, which can remove the interference of the inclination angle on the measurement accuracy and is applicable for dynamic deflection monitoring of measurement points with large height differences.

## 2. Basic Principle of LLSS-Based Deflection Measurement

**Figure 2:**
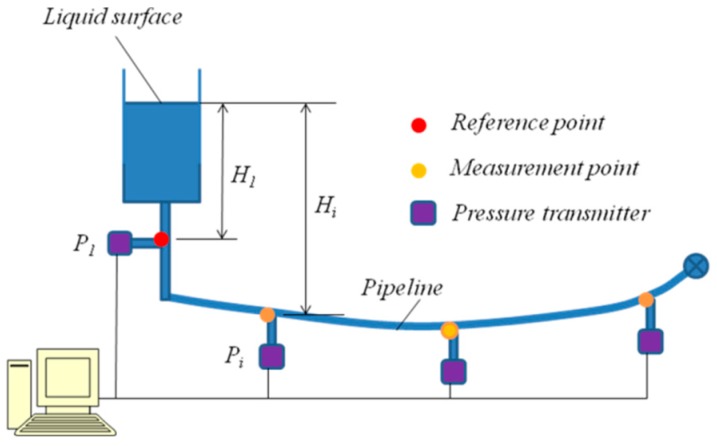
Layout of a typical liquid level sensing system.

The LLSS consists of pipelines, liquid, and pressure transmitters, as shown in Figure 2. The pipeline is deployed along the main girder of a bridge and filled with liquid (e.g., water). Pressure transmitters are arranged at the measurement points (*P_i_*), which are connected with the pipeline. In general, the reference point (*P*_1_) is set at the pier of the bridge or a fixed point near the river shore. When deflection appears under loads, the pressure changes at the measurement points caused by the changes in liquid level are captured by the pressure transmitters and then converted to deflection by algorithms.

The static deflection is obtained from Equation (1).

(1) $$u_{i,t}=\frac{\Delta P_{i,t}}{\rho g}=\frac{P_{i,t}-P_{1,t}}{\rho g}$$

where $u_{i,t}$ and $\Delta P_{i,t}$ represent the deflection and pressure change in the *ith* measurement point at *t* moment, respectively; $P_{i,t}$ and $P_{1,t}$ indicate the pressure of the *ith* measurement point at *t* moment; *ρ* depicts the density of the liquid; and *g* indicates gravitational acceleration.

**Figure 3:**
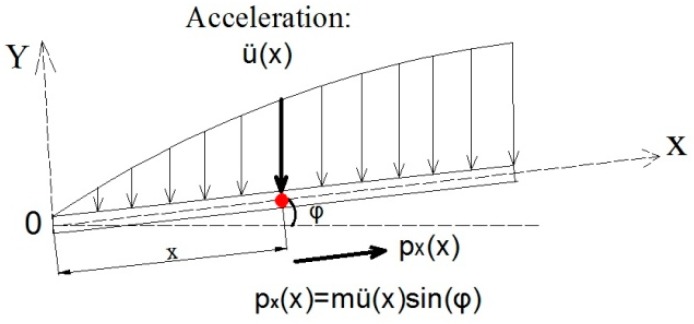
Acceleration distributed along the X-axis.

For the dynamic deflection measurement, along with the bridge structural vibration, the liquid in the pipeline is dynamic, and Equation (1) does not work for dynamic deflection derivation. The influence of the fluid motion on the pressure measurement accuracy has to be analyzed and corrected. Since the forced vibration of liquid would place additional pressure on the pipeline. As shown in Figure 3, the acceleration function distributed along the X-axis is defined as $\ddot{u}\left(x\right)$. Under the mode of vibration, the pipe will exert vertical pressure $p\left(x\right)=m\cdot \ddot{u}\left(x\right)$ on liquid in the pipeline and force the liquid to move synchronously with the pipe [36].

The acceleration component along the X-axis is

(2) $$p_{x}\left(x\right)=m\cdot \ddot{u}\left(x\right)\cdot \sin\left(\varphi \right)$$

At moment *t* (time = *t*), the pressure change of the *ith* measurement point can be expressed as

(3) $$\Delta P_{{}_{i,T}}^{a}=\frac{1}{A}\int_{x_{1}}^{x_{2}}{\ddot{u}}_{i,t}\left(x\right)\sin\left(\varphi \right)dm=\rho \sin\left(\varphi \right)\int_{x_{1}}^{x_{2}}{\ddot{u}}_{i,t}\left(x\right)dx$$

where *x*_1_ is the distance between the point 0 and the *ith* measurement point; *x*_2_ is distance between the point 0 and the center of the pipeline; *A* is the area of the pipe; and dm=Aρdx is the water mass of length *dx*. 

Pressure changes and acceleration data on measurement points must be collected synchronously to make dynamic deflection corrections. When the measured acceleration is substituted into Equation (4), additional pressure exerted due to structural vibration is obtained. The correction of dynamic deflection due to structural vibration and pipe inclination can be written as

(4) $$\Delta u_{{}_{i,t}}^{}=\frac{\rho \sin\left(\varphi \right)\int_{x_{1}}^{x_{2}}{\ddot{u}}_{i,t}\left(x\right)dx}{\rho g}$$

Hence, when combined with Equation (1), the total relative deflection can be expressed as

(5) $$u=u_{i,t}+\Delta u_{{}_{i,i}}^{}$$

In Equation (5), the first part denotes static deflection derived according to the static measurement principle of the LLSS; the second part denotes the correction of dynamic deflection deduced by considering additional pressure exerted due to structural vibrations.

## 3. Dynamic Characteristic of the LLSS Liquid

As noted above, the forced vibration of liquid in the pipeline occurs due to the influence of structural vibrations, which place additional pressure on the pipeline. Due to structural vibration, the fluid flows back and forth in the pipeline, transmitting a pressure signal during deflection monitoring. Therefore, the LLSS is a pulsating flow hydraulic (PFH) system, since the transmission efficiency of a PRH system greatly affects the dynamic characteristic of the system. In this section, fluid dynamics [38] is firstly used to introduce the influence of fluid velocity on pressure, then PRH [39] is applied to introduce the influence of transmission efficiency on dynamic characteristics of LLSS.

### 3.1. Fluid Dynamics of the Straight-Line-Type LLSS

#### 3.1.1. Equations of Fluid Dynamics

With the Euler method in fluid dynamics, the fluid motion in the pipeline obeys the continuity equation written in differential form as

(6) $$\frac{\partial \rho }{\partial t}+\frac{\partial \left(\rho u\right)}{\partial x}+\frac{\partial \left(\rho v\right)}{\partial y}+\frac{\partial \left(\rho w\right)}{\partial z}=0$$

where *ρ* means the density of liquid; and *u*, *v*, and *w* represent components of the flow velocity in the *x*, *y*, and *z* directions, respectively. The liquid between any two adjacent sections in the pipeline is considered to obey conservation of mass. In unit time, the mass of the inflow equals that of the outflow.

The momentum equation of fluid motion can be expressed as

(7) $$\left\{ \begin{array}{l} \rho \frac{Du}{Dt}=\rho F_{x}-\frac{\partial P}{\partial x}+\frac{\partial \tau_{xx}}{\partial x}+\frac{\partial \tau_{yx}}{\partial y}+\frac{\partial \tau_{zx}}{\partial z}\\ \rho \frac{Dv}{Dt}=\rho F_{y}-\frac{\partial P}{\partial y}+\frac{\partial \tau_{xy}}{\partial x}+\frac{\partial \tau_{yy}}{\partial y}+\frac{\partial \tau_{zy}}{\partial z}\\ \rho \frac{Dw}{Dt}=\rho F_{z}-\frac{\partial P}{\partial z}+\frac{\partial \tau_{xz}}{\partial x}+\frac{\partial \tau_{yz}}{\partial y}+\frac{\partial \tau_{zz}}{\partial z} \end{array}\right.$$

where $F_{x}$, $F_{y}$, and $F_{z}$ represent the components of force per unit mass in the *x*, *y*, and *z* directions, respectively; *P* means the pressure per unit mass; and $\tau_{ij}$ depicts the shear stress of fluid motion.

The substantial derivative of acceleration can be written as

(8) $$\left\{ \begin{array}{l} \frac{du}{dt}=\frac{\partial u}{\partial t}+u\frac{\partial u}{\partial x}+v\frac{\partial u}{\partial y}+w\frac{\partial u}{\partial z}\\ \frac{dv}{dt}=\frac{\partial v}{\partial t}+u\frac{\partial v}{\partial x}+v\frac{\partial v}{\partial y}+w\frac{\partial v}{\partial z}\\ \frac{dw}{dt}=\frac{\partial w}{\partial t}+u\frac{\partial w}{\partial x}+v\frac{\partial w}{\partial y}+w\frac{\partial w}{\partial z} \end{array}\right.$$

Combining Equations (6) and (7), Equation (8) can be expressed as

(9) $$\left\{ \begin{array}{l} \rho \frac{Du}{Dt}=\frac{\partial \left(\rho u\right)}{\partial t}+u\frac{\partial \left(\rho u^{2}\right)}{\partial x}+v\frac{\partial \left(\rho uv\right)}{\partial y}+w\frac{\partial \left(\rho uw\right)}{\partial z}\\ \rho \frac{Dv}{Dt}=\frac{\partial \left(\rho v\right)}{\partial t}+u\frac{\partial \left(\rho uv\right)}{\partial x}+v\frac{\partial \left(\rho v^{2}\right)}{\partial y}+w\frac{\partial \left(\rho vw\right)}{\partial z}\\ \rho \frac{Dw}{Dt}=\frac{\partial \left(\rho w\right)}{\partial t}+u\frac{\partial \left(\rho uw\right)}{\partial x}+v\frac{\partial \left(\rho vw\right)}{\partial y}+w\frac{\partial \left(\rho w^{2}\right)}{\partial z} \end{array}\right.$$

**Figure 4:**
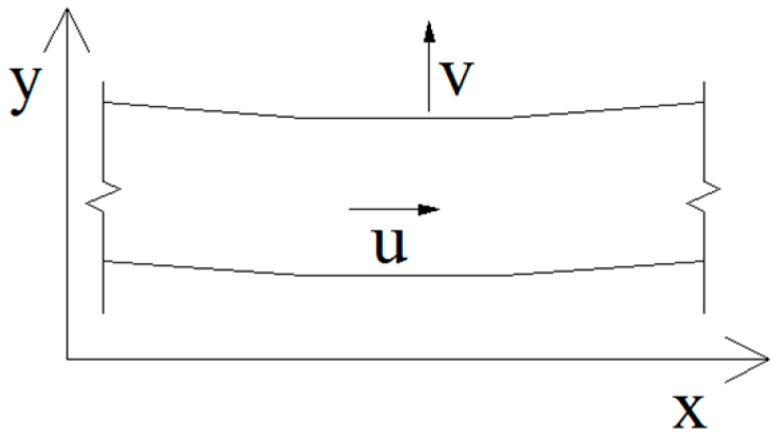
Two-dimensional fluid element.

As discussed above, the pressure at any point of the fluid not only relies on the position head of the point, but it also depends on the fluid velocity in the *x*, *y*, and *z* directions. The corresponding pressure change can be obtained when the fluid velocity at the point is given. Compared with the axial and vertical fluid velocities, the lateral velocity *w* is small and may be ignored. The fluid motion in the pipeline is considered to be two-dimensional, as shown in Figure 4.

Then, Equation (9) can be simplified as

(10) $$\left\{ \begin{array}{l} \rho \frac{Du}{Dt}=\frac{\partial \left(\rho u\right)}{\partial t}+u\frac{\partial \left(\rho u^{2}\right)}{\partial x}+v\frac{\partial \left(\rho uv\right)}{\partial y}\\ \rho \frac{Dv}{Dt}=\frac{\partial \left(\rho v\right)}{\partial t}+u\frac{\partial \left(\rho uv\right)}{\partial x}+v\frac{\partial \left(\rho v^{2}\right)}{\partial y} \end{array}\right.$$

The constitutive equations of fluid motion can be expressed as

(11) $$\left\{ \begin{array}{l} \tau_{xy}=\mu \left(\frac{\partial u}{\partial y}+\frac{\partial v}{\partial x}\right)=\tau_{yx}\\ \tau_{xx}=2\mu \frac{\partial u}{\partial x}-\frac{2}{3}\mu \left(\frac{\partial u}{\partial x}+\frac{\partial v}{\partial y}+\frac{\partial w}{\partial z}\right)\\ \tau_{yy}=2\mu \frac{\partial v}{\partial y}-\frac{2}{3}\mu \left(\frac{\partial u}{\partial x}+\frac{\partial v}{\partial y}+\frac{\partial w}{\partial z}\right) \end{array}\right.$$

Combining the continuity equation, momentum equation, and constitutive equation (Equations (6), (9), and (11)), the Navier–Stokes (N–S) equations can be obtained as

(12) $$\frac{\partial Q}{\partial t}+\frac{\partial F}{\partial x}+\frac{\partial G}{\partial y}=R$$

where

$$Q=\left(\begin{array}{l} \rho \\ \rho u\\ \rho v \end{array}\right),F=\left(\begin{array}{l} \rho u\\ \rho u^{2}\\ \rho uv-\tau_{xx} \end{array}\right),G=\left(\begin{array}{l} \rho v\\ \rho uv-\tau_{yz}\\ \rho v^{2}+P-\tau_{xy} \end{array}\right),R=\left(\begin{array}{l} 0\\ \rho F_{x}\\ \rho F_{y} \end{array}\right)$$

The fluid motion in the pipeline is turbulent with relatively divergent velocity. The Reynolds-derived Reynolds-averaged Navier–Stokes (RANS) equations based on a time-averaged velocity field [40,41] are shown as

(13a) $$\rho \left(\frac{\partial \bar{u}}{\partial t}+\bar{u}\frac{\partial \bar{u}}{\partial x}+\bar{v}\frac{\partial \bar{u}}{\partial y}+\bar{w}\frac{\partial \bar{u}}{\partial z}\right)=-\frac{\partial \bar{P}}{\partial x}+\mu \left(\frac{\partial^{2}\bar{u}}{\partial x^{2}}+\frac{\partial^{2}\bar{u}}{\partial y^{2}}+\frac{\partial^{2}\bar{u}}{\partial z^{2}}\right)+\frac{\partial \rho \bar{u^{\prime }u^{\prime }}}{\partial x}+\frac{\partial \rho \bar{v^{\prime }v^{\prime }}}{\partial y}+\frac{\partial \rho \bar{u^{\prime }w^{\prime }}}{\partial z}$$

(13b) $$\rho \left(\frac{\partial \bar{v}}{\partial t}+\bar{u}\frac{\partial \bar{v}}{\partial x}+\bar{v}\frac{\partial \bar{v}}{\partial y}+\bar{w}\frac{\partial \bar{v}}{\partial z}\right)=-\frac{\partial \bar{P}}{\partial y}+\mu \left(\frac{\partial^{2}\bar{v}}{\partial x^{2}}+\frac{\partial^{2}\bar{v}}{\partial y^{2}}+\frac{\partial^{2}\bar{v}}{\partial z^{2}}\right)+\frac{\partial \rho \bar{u^{\prime }v^{\prime }}}{\partial x}+\frac{\partial \rho \bar{v^{\prime }v^{\prime }}}{\partial y}+\frac{\partial \rho \bar{v^{\prime }w^{\prime }}}{\partial z}$$

(13c) $$\rho \left(\frac{\partial \bar{w}}{\partial t}+\bar{u}\frac{\partial \bar{w}}{\partial x}+\bar{v}\frac{\partial \bar{w}}{\partial y}+\bar{w}\frac{\partial \bar{w}}{\partial z}\right)=-\frac{\partial \bar{P}}{\partial z}+\mu \left(\frac{\partial^{2}\bar{w}}{\partial x^{2}}+\frac{\partial^{2}\bar{w}}{\partial y^{2}}+\frac{\partial^{2}\bar{w}}{\partial z^{2}}\right)+\frac{\partial \rho \bar{u^{\prime }w^{\prime }}}{\partial x}+\frac{\partial \rho \bar{v^{\prime }w^{\prime }}}{\partial y}+\frac{\partial \rho \bar{w^{\prime }w^{\prime }}}{\partial z}$$

**Figure 5:**
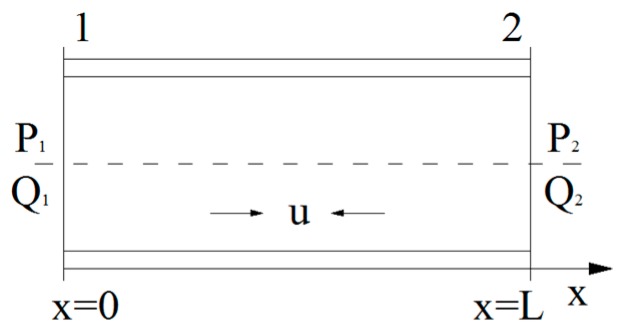
Fluid element of one dimension.

For turbulent flow, the fluid pressure at any point is closely related to time-averaged fluid velocity. $\bar{u}$, $\bar{v}$, and $\bar{w}$ are the time-averaged fluid velocities of the *x*, *y*, and *z* directions. $u^{\prime }$, $v^{\prime }$, and $w^{\prime }$ are the fluctuating velocity of the *x, y,* and *z* directions. Since the fluid model in this study can be approximately considered as a one-dimensional fluid element, as shown in Figure 5, only the effect of the time-averaged fluid velocity in the axial direction is studied.

#### 3.1.2. Pulsating Flow Hydraulic of a One-Dimensional Fluid Element

As the fluid motion in the pipeline is a turbulent flow, the distribution of axial fluid velocity (*v*) will be interfered with by the turbulent flow field. The pressure transmission efficiency has to be analyzed. In Figure 5, due to structural vibration, fluid flows back and forth in the pipeline, transmitting a pressure signal during deflection monitoring.

According to the flow field and pipeline characteristics of the pulsating flow hydraulic system, the flow volume and pressure of the fluid satisfy

(14) $$\left[\begin{array}{l} P_{2}\\ Q_{2} \end{array}\right]=\left[\begin{array}{l} G_{11}\;\;\;G_{12}\\ G_{21}\;\;\;G_{22} \end{array}\right]\left[\begin{array}{l} P_{1}\\ Q_{1} \end{array}\right]$$

where *P*_1_ and *Q*_1_ represent the pressure and flow volume of Section 1, respectively, shown in Figure 5; *P*_2_ and *Q*_2_ indicate the pressure and flow values of Section 2, respectively. The transmission efficiency of the pressure from Section 1 to Section 2 depends on the parameters G_11_, G_12_, G_21_, and G_22_,

(15) $$\left\{ \begin{array}{l} G_{11}=G_{22}=\cosh\left(\Gamma \right)l\\ G_{12}=-Z_{0}\sinh\left(\Gamma \right)l\\ G_{22}=-\sinh\left(\Gamma \right)l/Z_{0} \end{array}\right.$$

where Γ, Z_0_, and *l* represent the propagation factor, characteristic impedance, and transmission length of the pipeline, respectively. The transmission efficiency of the pressure between Section 1 and Section 2 of the pipeline can be obtained with

(16) $$\eta =\frac{P_{2}}{P_{1}}={\left(\cosh\left(\Gamma \right)l+Z_{2}Z_{0}\sinh\left(\Gamma \right)l\right)}^{-1}$$

where Z_2_ depicts the output impedance of the pipeline, including the output impedance of the press-leading tube and the pressure transmitter.

In Equation (16), the pressure transmission efficiency mainly relied on the transmission length, characteristic impedance, and output impedance of the pipeline. The transmission length (*l*) depends on the length of the structure to be monitored, and the characteristic impedance (Z_0_) of the pipeline depends on the physical characteristics of the pipeline itself. The output impedance (Z_2_) of the pipeline mainly relied on the output impedance of the pressure transmitter, which has a great influence on the dynamic characteristics of LLSS. As an intrinsic parameter of the instrument, the characteristic impedance of the pressure transmitter can be measured by testing. By a series of tests, Ye [36] concluded that the ROSEMOUNT 3051CD pressure transmitter has more than 90% pressure measurement accuracy when the basic frequency of the measured structure is less than 2 Hz.

### 3.2. Fluid Dynamics for the Proposed Step-Type LLSS

**Figure 6:**
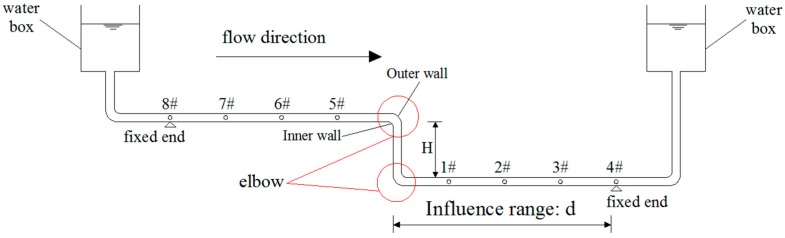
Layout of the step-type LLSS.

In Figure 6, when the liquid flows through the elbow, due to the centrifugal inertia force, the liquid will flow to the outer wall of the elbow, which causes the liquid to depart from the inner wall and form an eddy current zone near the inner wall. The pressure on the outer wall of the pipe rises, and the pressure on the inner wall of the pipe decreases. The diffusion effect is generated near the outer wall, and the convergence effect occurs near the inner wall. Due to the coupling effect of the centrifugal inertia force and boundary layer, a secondary flow exists inside the elbow. A spiral flow will exist by superimposing the secondary flow with the main stream of the pipe, which will disappear very slowly over a long distance. When the liquid flows through the pipeline, the loss of kinetic energy in the range of *d* happens, as shown in Figure 6. The resistance coefficient of the elbow not only relies on the Reynolds coefficient but also depends on the geometric parameters of the elbow, such as the bending angle and curvature radius. If the pressure transmitter is deployed in the range of *d*, the measurement accuracy will be affected.

The forced vibration of liquid in the pipeline, which occurs due to the influence of structural vibrations, will generate additional pressure along the pipeline at the measurement point [36]. According to fluid dynamics [38], the total pressure can be written as

(17) $$P+\frac{1}{2}\rho V^{2}=\rho gH$$

where *P* represents the static pressure of the measurement point; and *V* and $\frac{1}{2}\rho V^{2}$ mean the fluid velocity and additional dynamic pressure, respectively.

The additional pressure is ignored for static deflection measurement by LLSS. However, for dynamic deflection measurement, the deflection error $\Delta H_{1}$ will exist while ignoring the additional pressure, shown as

(18) $$\Delta H_{1}=\frac{V^{2}}{2g}$$

In Figure 6, the fluid flows from measurement point #5 to #1. The fluid velocities at measurement points #5 and #1 are defined as *V*_2_ and *V*_3,_ respectively. Without considering the pipeline friction effect, due to the loss of kinetic energy near the elbow, *V*_2_ > *V*_3_. The corresponding loss of dynamic pressure from points #5 to #1 is

(19) $$\Delta P=\frac{1}{2}\rho V_{2}{}^{2}-\frac{1}{2}\rho V_{3}{}^{2}=\rho gh_{j}=\zeta \frac{1}{2}\rho V_{3}^{2}$$

where $h_{j}$ means the local head loss, $h_{j}=\zeta \frac{V^{2}}{2g}$; and *ζ* depicts the local head loss coefficient of the circular bending pipe listed in Table 1. For straight-line-type LLSS, *V*_2_ = *V*_3_ means no dynamic pressure loss.

Through the above analysis, the flow field is redistributed near the elbow in the step-type LLSS, and the measurement accuracy of deflection will be affected. For the test model used in this paper, the parameters of the selected pipeline are as follows: circular-sectioned 90^0^ bend, *R*/*d’* = 2.0, *θ* = 90°, and *ζ* = 0.159. The corresponding loss of dynamic pressure is 15.9%, which is too large to be neglected. In the following sections, numerical simulation and experiments are applied to study the deflection measurement accuracy and reasonable arrangement of measurement points near the elbow.

## 4. Numerical Simulation

ANSYS-FLOTRAN is used for numerical simulation of the pressure field of turbulent flow in the elbow of the step-type LLSS. In the finite element model (FEM), the pressure distribution of each section and the influence range of turbulent flow are obtained.

The parameters of the FEM are as follows:

The elbow is a circular-sectioned 90º bend. The diameter of the elbow is *d’* = 0.05 m. The curvature radius of the inner wall and outer wall are *R_i_* = 0.1 m and *R*_0_ = 0.15 m, respectively. The radius ratio of the elbow is *R*/*d’* = 2.5, where R = (*R_i_* + *R*_0_)/2. 

The pipeline consists of three parts: the section of the lower straight line (length *l*_1_ = 10, *R* = 0.5 m), the section of the two elbows (length *l*_2_ = 10, *R* = 0.5 m) and the section of the upper straight line (length *l*_3_ = 30, *R* = 1.5 m).

The FLUID141 element is used. The model is meshed with 1126 grids and 1247 nodes. The meshing is dense in the section of the two elbows, while it is sparse in parts of the straight line section. 

Boundary conditions: The fluid is assumed to be incompressible, and its properties are constant. The nonslip boundary condition is applied to the walls of the pipeline (i.e., the velocity components are 0). The initial flow velocity condition of the inlet given by the FEM is a time-average liquid pressure (10.279 Pa). The initial air pressure of the inlet and outlet is taken as atmospheric pressure, which means that the relative pressure is 0. 

Water with a density of 1000 kg/m^3^ and kinematic viscosity of 1.136 × 10^−6^ m^2^/s is used as the liquid in the pipeline. The liquid flows from right to left, and the calculation is iterated 300 times.

**Figure 7:**
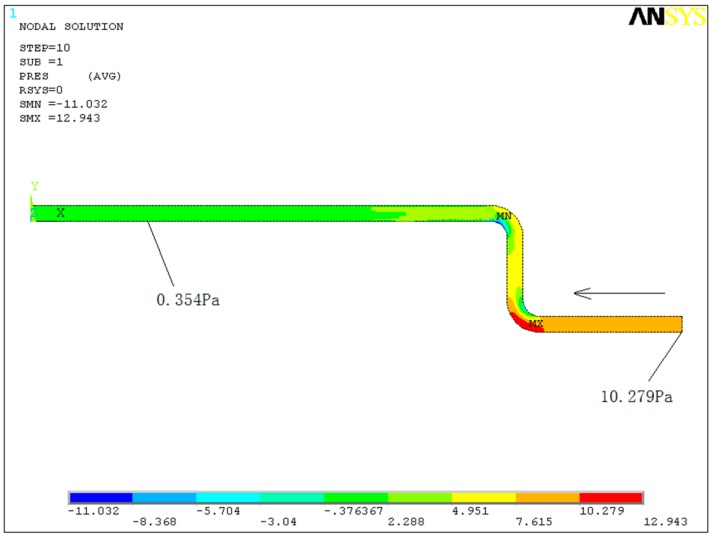
Pressure distribution in the numerical simulation.

In Figure 7, the pressure is disturbed by turbulent flow in the section of the elbows. For the elbow, the pressure of the outer wall is larger than that of the inner wall, and the pressure decreases when the fluid flows through the section of the two elbows, agreeing with the discussion in Section 3.2. When the fluid travels a distance (1.382 m) away from the upper elbow, the pressure becomes constant (0.354 Pa).

## 5. Experimental Verification

A test model was designed in the laboratory to study the dynamic characteristics of the step-type LLSS. Both the displacement meter and LLSS were applied to capture the dynamic deflection in distinct cases as verification.

### 5.1. Design of the Experimental Model

#### 5.1.1. Data Acquisition System

**Figure 8:**
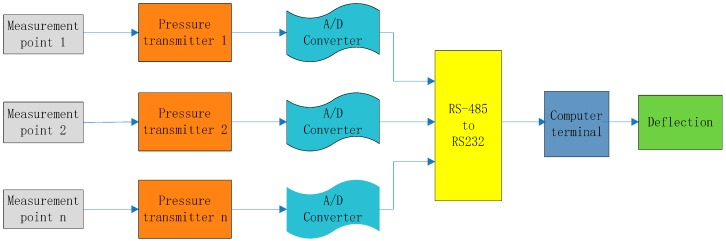
Data acquisition system.

In Figure 8, the data acquisition system consists of four modules: pressure transmitter, analogue-to-digital converter (A/D converter), RS-485 to RS-232 converter, and computer terminal. For deflection monitoring, the pressure change, detected by a pressure transmitter connected with the pipeline at the measurement point, was transferred to the converters and computer terminal, and then the pressure change was converted to deflection. 

**Figure 9:**
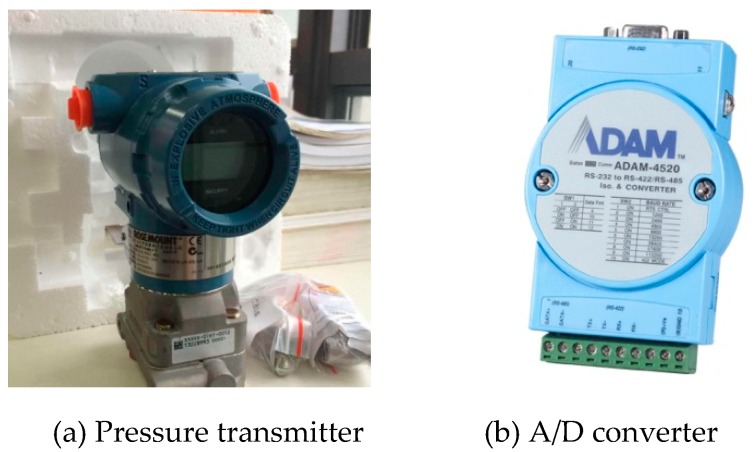
Pressure transmitter and A/D converter.

A ROSEMOUNT 3051CD pressure transmitter with a maximum sampling frequency of 4Hz was used in the LLSS. This type of transmitter has a resolution of 0.1 mm and a pressure measurement range of [0.12, 4.8] kPa (corresponding to a deflection measurement range of [0, 400] mm) [43]. Capacitance was used as the sensing element of the ROSEMOUNT 3051CD. When the pressure change was sensed by the sensing element, the capacitance changed, and the analogue signal of [1,5] volts or [4,20] mA was outputted. The ADAM-4520 converter, with a resolution of 16 bits, was used to convert the analogue signal to digital signal as shown in Figure 9. For the analogue signal of [4,20] mA, the resolution of the converter was 20/2^16^. As the maximum measurement range of the pressure transmitter is 4.8 kPa, the precision of the converter can reach 0.0011 mm.

#### 5.1.2. Design of the Structural Model

(1) Design Principle

**Figure 10:**
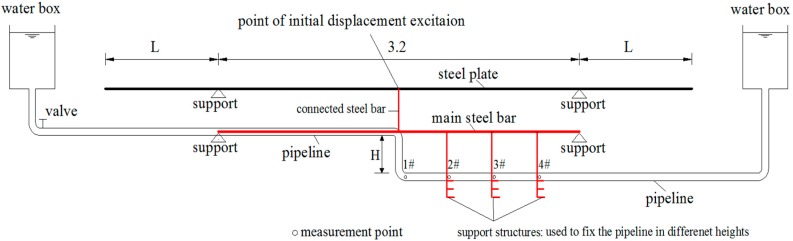
Design layout of the test model and step-type LLSS (unit: m).

In Figure 6, due to the turbulent flow caused by the elbow of the step-type LLSS, the measurement accuracy will be affected in the range of *d*, and the numerical simulation results demonstrate that *d* ≈ 1.382 m. With the elbow arranged in the midspan, the midspan of the test structure was chosen as 3.2 m, shown in Figure 10. As the measurement accuracy will be affected while the pipeline is deployed with an inclination angle, in this experiment, the inclination angle under self-weight and other loads was controlled to be less than 1º.

To accurately capture the dynamic characteristic of the structure, according to Nyquist–Shannon’s sampling theorem [44], the sampling frequency should be more than two times the value of the basic frequency of the measured structure. Since the maximum sampling frequency of the ROSEMOUNT 3051CD pressure transmitter is 4 Hz, the basic frequency of the structural should be designed to be less than 2 Hz. Additionally, as mentioned in Section 3.1.2, the pressure measurement accuracy using the ROSEMOUNT 3051CD pressure transmitter can be more than 90% when the basic frequency of the measured structure is less than 2 Hz. Therefore, the basic frequency of the test structure should be designed to be as small as possible, which was set as 1 Hz in this study.

(2) Structure design and FEM analysis

**Figure 11:**
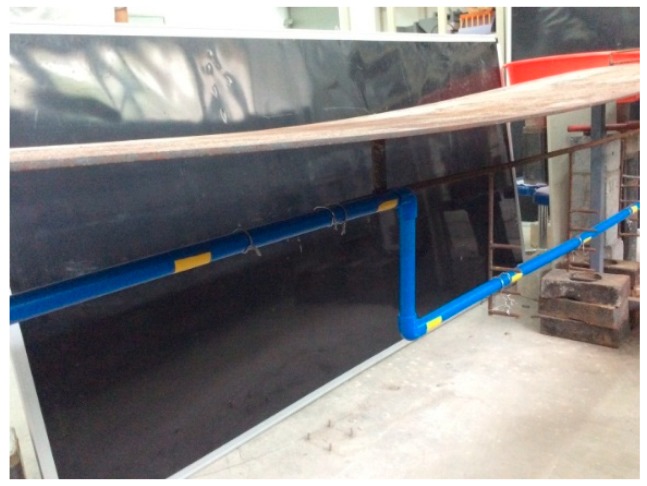
The test model and step-type LLSS in the laboratory.

Two key parameters for designing the structural model are the basic frequency and the inclination under self-weight and other loads. The steel plate has large flexibility (low basic frequency), and the steel bar has large stiffness (less deflection). After several comparisons and trials, a test structure, combining steel plate and steel bar, was applied as the main girder for the test model, shown in Figure 10 and Figure 11. The steel plate was designed as a cantilever structure with a midspan of 3.2 m and cantilever length of *L*. The pipeline was deployed along the main steel bar, a simply supported structure with a span of 3.2 m. The steel plate and main steel bar were connected in midspan by another steel bar. Additionally, the support structure was designed to fix the lower straight-line pipeline at different heights. 

In Table 2, six cases of different parameter combinations were designed, and Midas/Civil software determined the best parameter combinations for the test structure. With a midspan of 3.2 m, width of 0.5 *m*, and thickness of 5 *mm*, whether the cantilever length of the steel plate was 0 m or 1.0 m, the inclination angle was less than 1º, meeting the design principle. However, the basic frequency was larger than 1.24 Hz when the cantilever length was 0 m (cases 1, 3, and 5), while the basic frequency was less than 1.00 Hz when the cantilever length was 1.0 m (cases 2, 4, and 6). In comparison, the structure with both a lower basic frequency and smaller inclination angle was chosen as the test structure for experimental verification. Therefore, case 4 was chosen as the best parameter combination for the test model.

### 5.2. Experiment Description

As an improved dynamic deflection monitoring method, the measurement accuracy is of most concern. Two key parameters that might affect the measurement accuracy were studied in this paper; one is the step height, and the other is the value of the initial displacement excitation. Different situations of the experiment were designed to study the measurement accuracy of the step-type LLSS with different step heights under different initial displacement excitations.

An initial displacement excitation was applied in the midspan of the steel plate to excite the vibration of the structure. As shown in Table 3, three situations of initial displacement (P) were set as 15, 20, and 25 mm. In each situation, three cases of step height (H) were set as 0, 20, and 25 cm, respectively.

As mentioned before, the influence of turbulent flow on the liquid pressure distribution was the greatest at the elbow and decreased gradually along the pipeline. To analyze the influence range, four measurement points were deployed along the pipeline, shown in Figure 9. Measurement point #1 is set at the midspan, and the other measurement points are deployed every 0.5 m.

### 5.3. Parametric Analysis of the Measurement Accuracy

#### 5.3.1. Measurement Data Reliability

**Figure 12:**
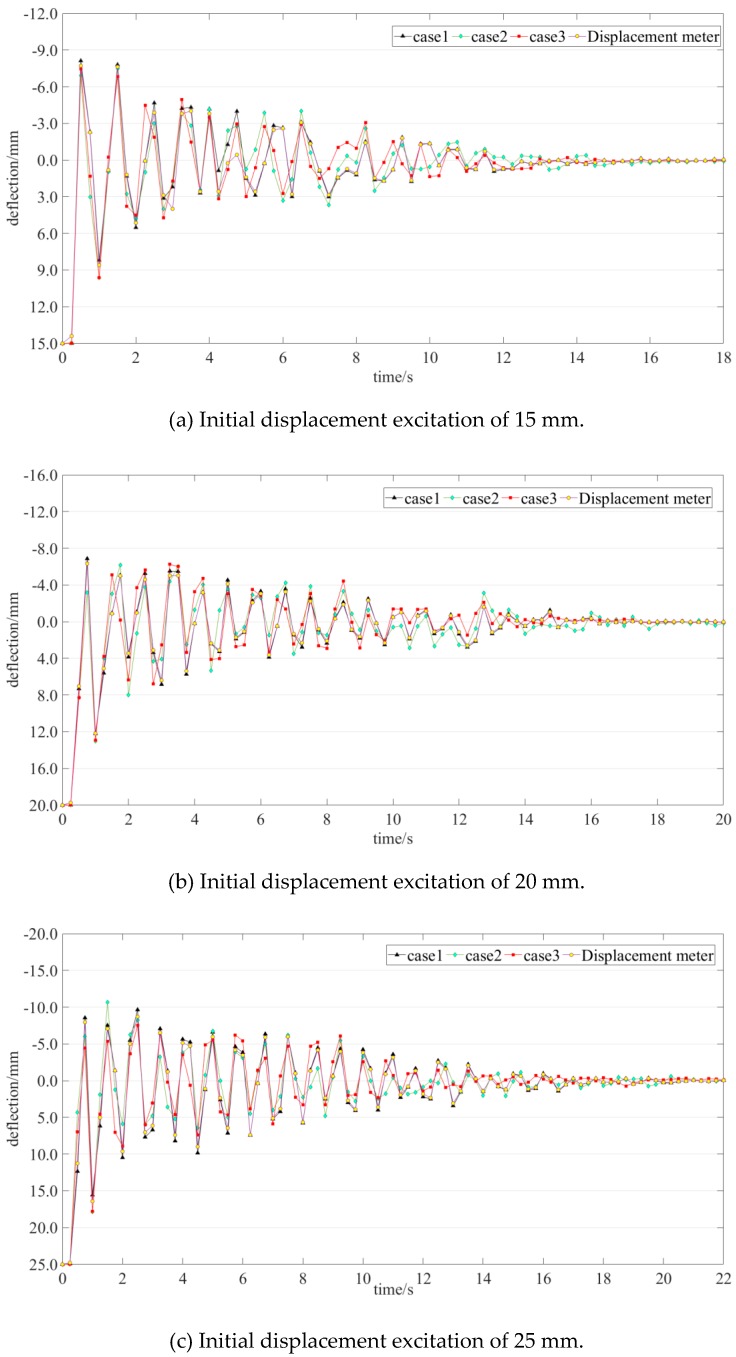
Deflection time history of measurement point #1 under different initial displacement excitations.

The deflection time histories of measurement point #1 under different initial displacement excitations are taken as an example. In Figure 12, the deflection at time 0 s indicates the value of the initial displacement. The four colored lines in each figure describe the data collected by the displacement meter and step-type LLSS with different step heights. The measurement data collected by the BJQN-V displacement meter were considered as a reference. With a measurement accuracy of ±0.02 mm (in the range of 10 m), BJQN-V is a contactless method that can simultaneously record lateral and vertical displacements of structures [45]. The correlation coefficients of the time history data between different cases and the reference are listed in Table 4. The correlation coefficients of the nine cases are larger than 0.94, which means that the data collected by the step-type LLSS are reliable. 

#### 5.3.2. Influence of the Initial Displacement Excitation and Step Height on the Measurement Accuracy

The largest value of the time history of the dynamic deflection is an important index, reflecting the deflection caused by heavy vehicles, and was chosen for further study. As shown in Figure 12, the deflection at time 0 s indicates the value of the initial displacement excitation, and the value of the first valley in the time history was the largest. For measurement point #1, the measured data from the displacement meter were taken as a reference; the measured values of the first valley and measurement errors of all situations are listed in Table 5.

**Figure 13:**
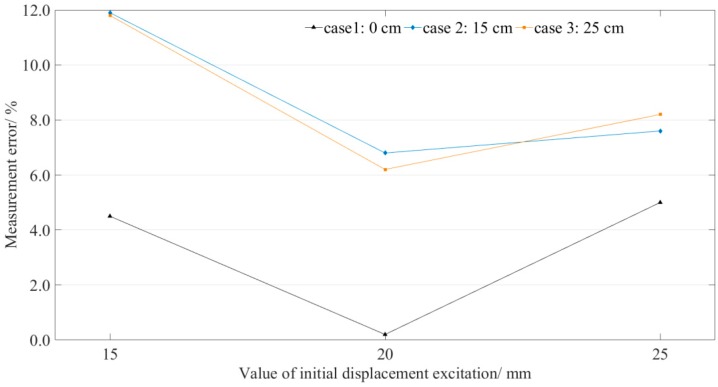
Measurement point #1: measurement errors distribution.

The measurement errors for point #1 are illustrated in Figure 13. For each case (the step height is constant), the measurement error does not increase with the increase in the excited initial displacement. The results show no obvious correlation between the measurement errors and the value of initial displacement. However, in each situation (the value of initial displacement excitation is constant), the measurement error increases with the increase in the step height. When the step was introduced in the LLSS, all the measurement errors of measurement point #1 (position of the elbow) were more than 6%, which is unacceptable. Therefore, the measurement point should be deployed away from the elbow. 

#### 5.3.3. Influence Range along the Pipeline

**Figure 14:**
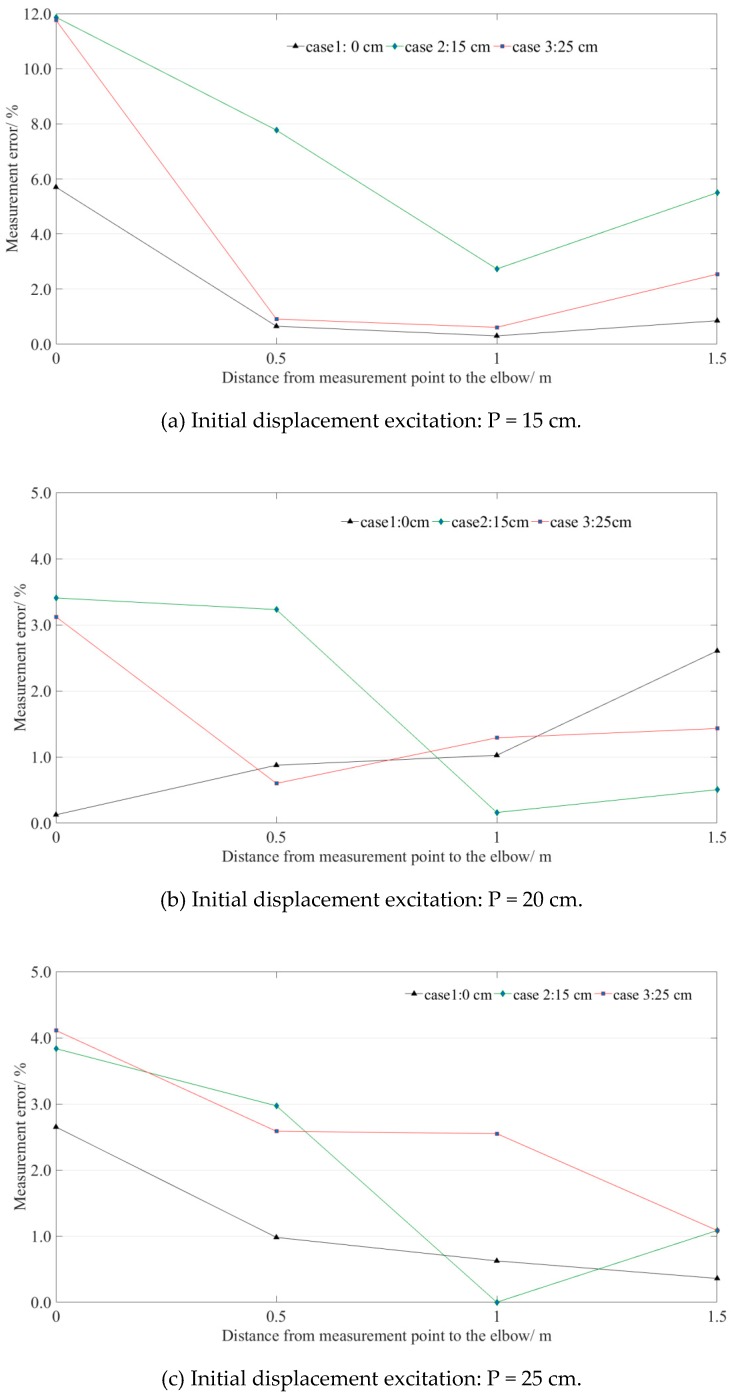
Measurement errors of the four measurement points distributed along the pipeline.

Four measurement points were deployed along the pipeline, and the measurement errors of all the situations are illustrated in Figure 14. The measurement errors of all measurement points were under 6% when the step height was 0 cm. That means the measurement point can be deployed at any position of the straight-line-type LLSS (H = 0 cm). For the step-type LLSS, the measurement error decreases as the distance away from the measurement point to the elbow increases. The measurement errors were distributed from 0% to 6% when the distance from the measurement point to the elbow was in [1.0, 1.5] m, satisfying the engineering requirement. 

**Figure 15:**
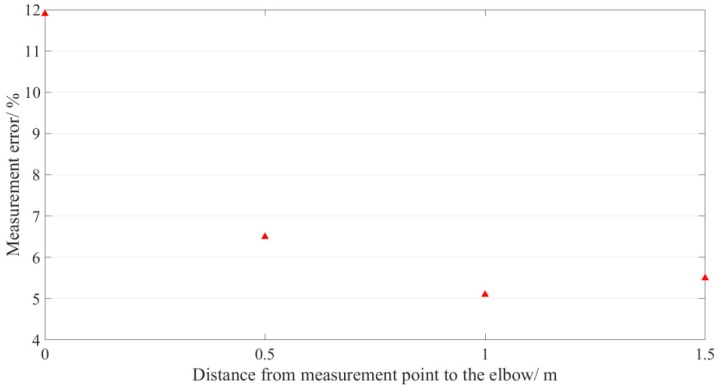
Fitting curve of the maximum errors of the four measurement points.

The maximum errors of the four measurement points in all situations are illustrated in Figure 15. The measurement error gets smaller after about 1.0 m distance from the elbow. To ensure the measurement error (*y*) of the measurement point is less than 6%, the distance (*x*) between the measurement point and the elbow should be greater than 1.0 m. 

## 6. Conclusions

This study illustrated an improved step-type LLSS for dynamic deflection monitoring. Numerical simulation and experimental verification were carried out to validate the reliability and measurement accuracy of the proposed method. Some findings are concluded as follows:

Whether the pipeline of LLSS was deployed in straight-line-type or step-type, no obvious relationship between the initial displacement excitation and measurement error was found. The measurement error does not increase as the excited initial displacement increases. The measurement accuracy was mainly interfered with by turbulent flow near the elbow. With the same value of initial displacement excitation, the measurement error increases as the step height near the elbow increases. The measurement error decreases with the increase in the distance away from the measurement point to the elbow. To ensure the measurement error is less than 6%, the distance between the measurement point and the elbow should be larger than 1.0 m. The proposed method is reliable and feasible for dynamic deflection monitoring. 

Only two key parameters (step height and the value of the initial displacement excitation) that might affect the measurement accuracy were studied in this paper. However, the measurement accuracy would be disturbed by environmental conditions and some system errors during installation (e.g., air bubbles in the pipeline, leakages) in field application. Further research on measurement accuracy interfered with by more complex conditions should be carried out before outdoor application. 

## Figures and Tables

**Table 1:** The local head loss coefficient of circular bending pipes [42].

Section Shape	*R/d’*	Bending Angle (*θ*)			
		30°	45°	60°	90°
**Circular**	0.5	0.120	0.270	0.480	1.000
	1.0	0.058	0.100	0.150	0.246
	2.0	0.066	0.089	0.112	0.159

Notes: *R* and *d’* are the curvature radius and diameter of the elbow, respectively.

**Table 2:** Result of the finite element model (FEM) from six cases of different parameter combinations.

Case	Parameter Combination					Result of FEM	
	Main Steel Bar Diameter (mm)	Steel Plate					
		Width (m)	Thickness (mm)	Span (m)	Cantilever Length (m)	Basic Frequency (Hz)	Inclination Angle
**1**	12	0.5	5	3.2	0	1.24	0.786
**2**	12	0.5	5	3.2	1.0	0.93	0.772
**3**	14	0.5	5	3.2	0	1.27	0.544
**4**	14	0.5	5	3.2	1.0	0.96	0.531
**5**	16	0.5	5	3.2	0	1.33	0.409
**6**	16	0.5	5	3.2	1.0	0.99	0.404

**Table 3:** Parameter settings.

Cases (Step Height: H)	Situations (Initial Displacement: P)		
	#1: P = 15 mm	#2: P = 20 mm	#3: P = 25 mm
**Case #1**	H = 0 cm	H = 0 cm	H = 0 cm
**Case #2**	H = 20 cm	H = 20 cm	H = 20 cm
**Case #3**	H = 25 cm	H = 25 cm	H = 25 cm

**Table 4:** Correlation coefficients between different cases and the reference.

Cases (Step Height)	Situations (Initial Displacement)		
	#1: *P* = 15 mm	#2: *P* = 20 mm	#3: *P* = 25 mm
**Case #1: *H* = 0 cm**	0.95	0.96	0.96
**Case #2: *H* = 20 cm**	0.94	0.95	0.94
**Case #3: *H* = 25 cm**	0.94	0.95	0.94

**Table 5:** Measurement point #1: values of the first valley of the time history data of all situations.

Cases (Step Height)	Situations (Initial Displacement)								
	#1: P = 15 mm			#2: P = 20 mm			#3: P = 25 mm		
	Deflection (mm)		Error (%)	Deflection (mm)		Error (%)	Deflection (mm)		Error (%)
	LLSS	DM		LLSS	DM		LLSS	DM	
**Case #1: H = 0 cm**	8.19	8.59	4.5	12.21	12.18	0.2	15.59	16.42	5.0
**Case #2: H = 20 cm**	9.61		11.9	13.01		6.8	17.68		7.6
**Case #3:H = 25 cm**	9.60		11.8	12.94		6.2	17.77		8.2

Notes: DM means displacement meter.
